# Inequalities in lesbian, gay, bisexual, and transgender (LGBT) health and health care access and utilization in Wisconsin

**DOI:** 10.1016/j.pmedr.2019.100864

**Published:** 2019-04-01

**Authors:** Linn Jennings, Chris Barcelos, Christine McWilliams, Kristen Malecki

**Affiliations:** a Department of Population Health Sciences, University of Wisconsin-Madison, Wisconsin Alumni Research Foundation, Madison, WI, USA; b Department of Gender and Women's Studies, University of Wisconsin-Madison, Madison, WI, USA

**Keywords:** LGBT health, Health care access, Health care utilization, Health disparities

## Abstract

There are known health disparities between lesbian, gay, bisexual and transgender (LGBT) people and non-LGBT people, but only in the past couple of decades have population-based health surveys in the United States included questions on sexual and gender identity. We aimed to better understand LGBT disparities in health, health care access and utilization, and quality of care. Data are from the Survey of the Health of Wisconsin (SHOW) from 2014 to 2016 (*n* = 1957). The analyses focused on comparing health care access and utilization, and quality of care between LGB and non-LGB people and transgender and cisgender people. 3.8% (*n* = 73) identified as lesbian, gay or bisexual, and 1.3% (*n* = 25) were transgender. LGB adults were 2.17 (95th CI: 1.07–4.4) times more likely to delay obtaining health care. Transgender adults were 2.76 (95th CI: 1.64–4.65) times more likely to report poor quality of care and 2.78 (95th CI: 1.10–7.10) unfair treatment when receiving medical care. The results show differences in health care access and utilization and quality of care, and they add to the growing body of literature that suggest that improved health care services for LGBT patients are needed to promote health equity for LGBT populations.

## Highlights

• LGBT individuals were more likely to report having fair/poor health than non-LGBT.
• LGB individuals were more likely to delay health care than non-LGB individuals.
• Trans respondents were more likely to report poor quality of care than non-LGBT.

## 1 Introduction

Health care access and utilization and quality of care are continuing to improve in the United States, but these improvements are not consistent across states or populations (Agency for Healthcare Research and Quality, 2016). Health promotion initiatives in the United States, such as the Center for Disease Control's (CDC) Healthy People initiatives, have focused on understanding how these other factors differ among various populations in order to improve population health outcomes (Alencar Albuquerque et al., 2016). These initiatives specifically target the health outcomes of marginalized groups, a term used to both define and understand the political and social impact of excluding and denying groups of people access to rights and services that are guaranteed to the rest of a country or society. These groups are at a higher risk of having low socioeconomic status and poor health outcomes, which contribute to why health disparities persist between marginalized and non-marginalized populations (Agency for Healthcare Research and Quality, 2016; Blosnich et al., 2014).

Despite known health disparities between marginalized and non-marginalized populations, until recently, LGBT populations were rarely recognized as marginalized populations requiring research focus in national health initiatives (Boehmer, 2002). Until the 2000's there were few surveys that included questions about sexual orientation and gender identity (Bradford et al., 2013). There are vast inconsistencies in the questions on sexual and gender identity, and few use the validated questions recommended by the William's Institute: (Bradford et al., 2013) three questions to establish sexual orientation (self-identification of sexual orientation, sexual behavior, and sexual attraction) (Braveman et al., 2010), and a validated two-step question approach to measuring gender identity for population-based surveys (sex-assigned at birth and gender identity) (Centers for Disease Control and Prevention, 2017). This incommensurability prevents surveys from identifying LGBT people with high sensitivity and specificity (Cohen, 2017), which limits our ability to estimate the size of these populations, understand the health disparities, and to address these disparities on an individual, health system, and policy level.

### 1.1 LGBT health disparities and why they exist

The BRFSS (Behavior Risk Factor Surveillance System) and NHIS (National Health Interview Survey) survey a nationally representative sample of the United States population, and they are widely used in developing health policies at all levels of government across the United States. Previous results from national population-based studies identified several factors contributing to LGBT health disparities, including discrimination and stigma (Conron et al., 2010; Cornelius and Carrick, 2015); limited access to health insurance (Cruz, 2014); poor quality of care provided due to both discrimination based on sexual orientation and gender identity (Conron et al., 2010; Durso and Meyer, 2013); lack of provider knowledge about LGBT health care needs (Gates, 2014); and insufficient research about the health of LGBT populations (Gorman, 2016; Graham et al., 2011). LGBT disparities in physical and mental health, health behaviors, and overall health status are shown to be linked to minority stress associated with the stigma and discrimination from having a minority status (Grant et al., 2010).

Despite increased awareness about LGBT health disparities and known causes of these disparities, which are established by several decades of research, limited regional and state-level population-based data about LGBT populations continues to act as a barrier to understanding LGBT health disparities in the United States (Bradford et al., 2013; Gorman, 2016; Graham et al., 2011). Health experiences of the LGBT community can vary by state and municipality due to differences in anti-discrimination laws and policies (Green et al., 2018; Hasenbush et al., 2014). Additional evidence based research at all levels is needed to inform policies aimed at understanding the impact of these systemic biases. One of the first steps to addressing these gaps is through research to better understand LGBT health disparities in health outcomes and health care access and utilization, which can then be used to inform policy and improve provider training.

### 1.2 Study aims

The Survey of the Health of Wisconsin (SHOW) program, a unique state-specific population-based research infrastructure, offers an important opportunity to study LGBT health care access and utilization. Unlike other population-based health surveys (like BRFSS and NHIS) that include few questions on health insurance status and health care utilization, SHOW includes extensive questions on health care access and utilization, which help provide insight into how LGBT population use health care services differently than non-LGB/cisgender populations.

This study had two primary aims: (Agency for Healthcare Research and Quality, 2016) to describe the LGB and transgender demographics, socioeconomic status, and occupation, and to compare these measure between LGB to non-LGB adults and transgender to non-LGB/cisgender adults in Wisconsin, and (Centers for Disease Control and Prevention, 2017) to analyze the differences between LGB adults and non-LGB adults and between transgender and non-LGB/cisgender adults in physical and mental health, discrimination in the medical setting, health care access and utilization, and quality of care.

## 2 Methods

SHOW is an annual household-based survey that collects health-related data on a representative population in Wisconsin. The sampling strategy for 2014–2016 used a three-stage cluster sampling approach to randomly select households, using a population-weighted proportion to size with replacement (PWPPSWR) sampling protocol. First, counties were sampled based on mortality rate, and then census blocks within counties were chosen based on poverty, and third, households were randomly sampled within census blocks. The three-year sample included Milwaukee and Dane counties (the two most populated counties in the state); ten counties in total were sampled.

Since 2014, SHOW asks questions on sexual orientation and gender identity (Table 1). These questions were chosen based on questions recommended by the Williams Institute and Fenway Health, but SHOW uses questions that are more similar to those used by BRFSS rather than the most recent best practice questions recommended by the Williams Institute (Braveman et al., 2010; Cohen, 2017).

**Table 1:** Survey of the Health of Wisconsin questions on sexual orientation and gender identity were asked during the duration of the study data (2014–2016). Questions were chosen based on the questions recommended by the Williams Institute and Fenway Health.

n	Response rate (%)	Sexual orientation and gender identity questions	Response options
1957	97.4	Do you consider yourself to be heterosexual or straight, gay or lesbian, or bisexual?	Heterosexual or straight; gay or lesbian; bisexual; don't know.
1957	97.2	Currently or in the past, have you identified as transgender, transsexual, or intersex?	Yes; no; don't know.
1957	94.1	If respondent answered yes or don't know to identifying as transgender, transsexual, or intersex they are asked this question: Some people describe themselves as transgender when they experience a different gender identity from their sex at birth. For example, a person born into a male body, but who feels female or lives as a woman. Which of the following describes you best?	Transgender female-to-male; Transgender male-to-female; Transgender not exclusively male or female, that is, I was born as female or male, but now I think of myself as neither male nor female; None of the above describes me.
1957	94.1	If respondent answered yes or don't know to identifying as transgender, transsexual, or intersex they are asked this question: Intersex is defined as being born with a body that is not exclusively male or female. Some people who are born as intersex end up thinking of themselves as male or female. Which of the following statements describes you best?	I was born as intersex, and now I consider myself male; I was born as intersex, and now I consider myself female; I was born as intersex, and now I do not consider myself exclusively male or female; None of the above describes me.

The SHOW data from 2014 to 2016 includes 1957 respondents who are age 18 and older. Of the survey respondents, 51 (2.6%) respondents did not answer the gender identity question, and 55 (2.8%) respondents did not answer the sexual orientation question. Of those who did not answer questions on sexual or gender identity, 41 (2.1%) respondents did not answer either of those questions.

### 2.1 Definitions

LGB included adults who identified as lesbian, gay or bisexual, and it included cisgender and transgender respondents. Cisgender is a term used for a person who identifies as their sex assigned at birth. Transgender is a term used for a person whose gender identity differs from their sex assigned at birth. Transgender included adults who identified as transgender or transsexual (intersex was removed from the comparison due to having a small n), and this measure included individuals who identified as LGB and non-LGB. The comparison group used for LGB and transgender was non-LGB/cisgender, which included adults who identified as heterosexual and were not transgender, transsexual or intersex. Occasionally LGBT was used to refer to both the LGB and transgender respondents.

### 2.2 Measures

Demographic variables included gender (for LGB and non-LGB only), age, race, occupation, income, education, and insurance status. Gender was excluded from the transgender analyses because it was unclear from the questions whether the transgender respondents answered the question about gender as sex-assigned at birth or as current gender identity. Occupation status was determined based on occupation status in the past week, income was assessed using the individual's income midpoint, and insurance status is measured by whether the respondent is currently insured.

Mental and physical health was assessed using the three measures: lifetime chronic illnesses, PHQ-2 depression screener score, Depression Anxiety Stress Scales (DASS) 21 short form scale. PHQ-2 is used as an assessment measure of depressive disorder, and it is not a diagnosis of depression. Scores range from 0 to 6, and scores of 3 and above have the highest sensitivities and specificities for identifying individuals with depressive disorders (Igartua et al., 2009). DASS scores are considered a good a measure of the constructs of depression, anxiety and stress and an overall measure of emotional distress (Ingham et al., 2018). We used the DASS21 z-score of 2.0 (moderate stress, anxiety, and depression) as our cut-point (Ingham et al., 2018).

Health behaviors assessed were cigarette smoking and drinking habits. Cigarette smoking was divided into two categories: current smoker and former and/or never smoker. Drinking habits were divided into two categories: Heavy drinker (>14 drinks per week for men or more than drinks per week for women) and light drinker (less than these two cut-off values).

The analysis also includes measures of health care access and utilization, quality of care and discrimination: self-report of frequency of use of primary care, whether the respondent usually sees the same physician, use of preventative care services, satisfaction and quality of care received by providers, lifetime discrimination and discrimination experienced when receiving medical care.

### 2.3 Statistical analysis

Multiple logistic regression and multiple linear regression analyses were used to assess the relationship between LGB and non-LGB/cisgender respondents and transgender and non-LGB/cisgender respondents in Wisconsin. The analyses for LGB, transgender, and non-LGB/cisgender respondents were adjusted for age, gender (only LGB and non-LGB/cisgender respondents), race, income, and education, which were chosen based on previous survey data analyses of these populations (Institute of Medicine (US) Committee on Lesbian, Gay, Bisexual, and Transgender Health Issues and Research Gaps and Opportunities, 2011). All statistical analysis was performed using SAS version 9.4 (SAS Institute Inc., Cary, North Carolina, USA) and weighted to adjust for sampling design. Sampling weights are generated for each data point by SHOW, and they are used to make the sample representative of the target population by stratifying by county, census block group, poverty, sex and race/ethnicity. The cluster, strata, and primary sampling unit estimates were used in the models run in SAS to calculate state-level estimates.

## 3 Results

### 3.1 Demographic and socioeconomic characteristics

Table 2 shows the demographic and socioeconomic characteristics of LGB adults (*n* = 73) compared to non-LGB/cisgender adults (*n* = 1830) and transgender adults (*n* = 25) compared to non-LGB/cisgender adults (*n* = 1830). Socioeconomic variables were adjusted for age and gender. The weighted prevalence of lesbian, gay or bisexual respondents was 3.8% (95% CI: 2.91–4.67), and the weighted prevalence of transgender respondents was 1.33% (95% CI: 0.7–1.95). There were several differences in demographics and socioeconomic status between LGB and non-LGB/cisgender adults; the mean age of LGB adults in the sample was younger than non-LGB/cisgender adults (*p* < 0.001), fewer were married or have partners compared to non-LGB/cisgender adults (*p* < 0.001), more lived below the 200% FPL (Federal Poverty Level) than non-LGB/cisgender adults (*p* = 0.023), and more were unemployed compared to non-LGB/cisgender adults (*p* = 0.058). The mean age of transgender adults (*n* = 25) was older than non-LGB/cisgender adults (*p* = 0.03), and no transgender adults in the sample were uninsured.

**Table 2:** Demographic characteristics for LGB (*n* = 73), transgender (*n* = 25), and heterosexual/cisgender adults (*n* = 1830) in Wisconsin from 2014 to 2016. Education, income, employment, insurance and marital status *p*-values were adjusted for age and gender for the comparison of LGB and heterosexual/cisgender adults and adjusted for age for the comparison of transgender and heterosexual/cisgender adults.

Characteristics	LGB *n* = 73	Transgender *n* = 25	Cisgender and heterosexual *n* = 1830
	3.84%	1.33%	96.16%
Mean age (se)	41.49(2.15)⁎	57.72(2.81)⁎	50.98(0.98)
Race:			
White	54 (79.9%)	22(95.03%)	1564(86.7%)
Black	6(5.21%)	1(1.82%)	115(5.2%)
Biracial	10(13.1%)	1(3.14%)	103(5.75%)
Education:			
High school or less	23 (43.7%)	8(29.6%)	454(31.3%)
Some college	7(11.7%)	3(10.0%)	304(21.5%)
College degree and above	21 (44.5%)	11 (60.4%)	688 (47.1%)
Location: urban	58(75.7%)	14 (53.2%)	1221(69.5%)
Below 200% FPL	33 (50.3%)⁎	7 (28.3%)	486 (28.9%)
Unemployed	27 (39.5%)	11 (36.6%)	750 (36.9%)
Insurance:			
Employment	41(63.9%)	14(62.4%)	990(62.4%)
Private/health exchange	12(16.7%)	5(13.6%)	296(14.9%)
Medicaid	13(18.8%)	1(3.01%)	268(15.9%)
Medicare	12(14.2%)	13(44.8%)	485(22.1%)
Uninsured	7 (8.9%)	0 (0%)⁎	121(7.6%)
Married/partner	23 (31.7%)⁎	18 (66.1%)	1172 (66.6%)
Divorced/separated/widowed	14(18.8%)⁎	3(9.1%)	320(15.3%)
Never married	35(49.5%)⁎	4(24.8%)	305(18.1%)

⁎ *p* < 0.05 compared to the cisgender/heterosexual group.

### 3.2 Health status

**Fig. 1:**
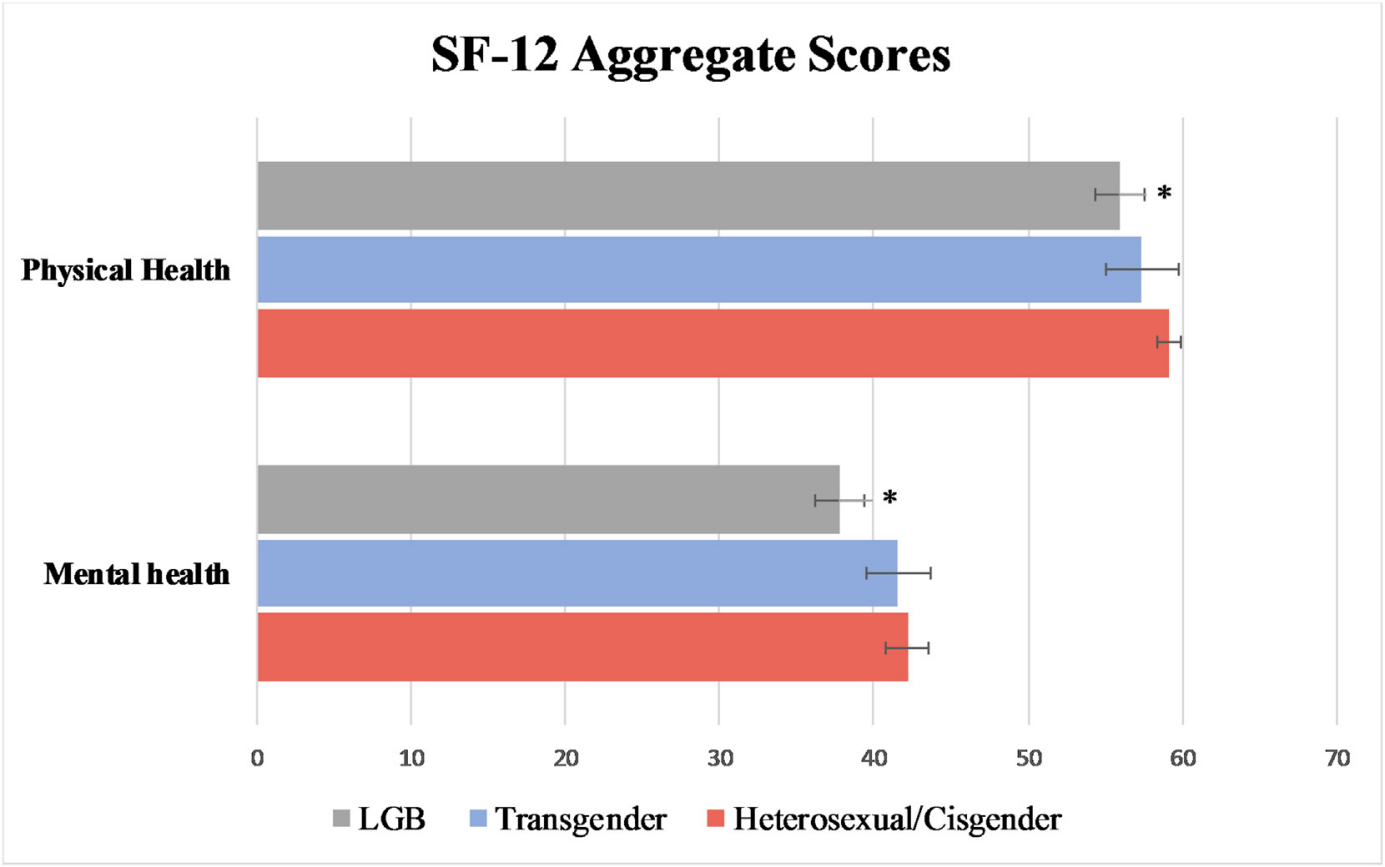
Aggregate scores on mental and physical health from the SF-12 for LGB compared to non-LGB/cisgender (adjusted for age and gender) and for transgender compared to non-LGB/cisgender (adjusted for age) in Wisconsin from 2014 to 2016. *p < 0.05.

Table 3 presents results about health status and health behaviors, and the indicators were adjusted for by age and gender. LGB adults reported fair or poor health more often than non-LGB/cisgender adults (OR:2.12, 95% CI: 0.95–4.73), were more likely to have a depression diagnosis based on PHQ-2 (OR:2.13, 95% CI:1.26–3.62), and more likely to have a moderate to severe depression score (OR: 2.59, 95% CI: 1.15–5.83) and anxiety score (OR:1.73, 95% CI:0.99–2.99). As shown in Fig. 1, LGB adults also scored lower on the SF-12 aggregate summary measures of mental (*p* = 0.049) and physical health (*p* < 0.01). Transgender respondents reported fair or poor health (OR: 2.22, 95% CI: 1.34–3.7), having a moderate to severe anxiety score (OR: 2.26, 95% CI:0.85–6.03), and having a history of chronic illness (OR:1.99, 95% CI: 0.86–4.6) more often than non-LGB/cisgender adults.

**Table 3:** Adjusted odds ratios for health indicators for LGB (*n* = 73) compared to non-LGB/cisgender (*n* = 1830) (adjusted for age and gender) and for transgender (*n* = 25) compared to non-LGB/cisgender (*n* = 1830) (adjusted for age) in Wisconsin from 2014 to 2016.

Health indicators	Odds ratio: LGB compared to non-LGB/cisgender (*n* = 73; n = 1830)	Odds ratio: transgender compared to non-LGB/cisgender (*n* = 25; *n* = 1830)	Percent of each group with the health indicator of interest: LGB; transgender; non-LGB/cisgender
Reported fair/poor health vs reported good/very good/excellent health	2.12 (0.95–4.73)+	2.22(1.34–3.7)⁎	22.22%; 26.09%; 1.94%
DASS depression z-score at or above 2.0 (moderate–extremely severe) vs below DASS depression z-score of 2.0	2.59(1.15–5.83)⁎	1.05(0.25–4.94)	23.29%; 8.00%; 9.65%
DASS stress z-score at or above 2.0 (moderate–extremely severe) vs below DASS stress z-score of 2.0	1.99(0.84–4.77)	0.55(0.07–4.15)	13.70%; 4.00%; 5.93%
DASS anxiety z-score at or above 2.0 (moderate–extremely severe) vs below DASS anxiety z-score of 2.0	1.73(0.99–2.99)⁎	2.26(0.85–6.03)+	17.81%; 20.00%; 9.71%
PHQ2 depression diagnosis (equal to or above 3) vs PHQ2 score below 3	2.13(1.26–3.62)⁎	1.66(0.28–9.8)	19.44%; 8.85%; 12.00
Chronic illness vs. no chronic illness	1.06(0.69–1.63)	1.99(0.86–4.6)	41.10%; 64.0%; 45.81%
Asthma diagnosis vs. no asthma diagnosis	1.61(0.83–3.1)	1.88(0.67–5.28)	19.8%; 20.00%; 11.11%
Heavy drinker vs light drinker	0.49(0.56–1.57)	0.9(0.17–4.74)	6.78%; 13.04%; 13.12%
Current smoker vs former/non-smoker	1.64(0.82–3.29)	0.47(0.09–2.13)	27.27%; 4.34%; 13.22%

⁎ *p* < 0.05.

+ *p* < 0.10.

### 3.3 Health care access and utilization

Table 4 shows the adjusted odds for indicators of health care access and utilization. LGB adults were more likely not to have the cost of preventative services covered by their insurance (OR:1.89, 95% CI:1.1–3.23), to delay obtaining needed health care (OR:2.17 95% CI:1.07–4.4), and to take less medicine than prescribed, (OR:2.14, 95% CI:0.82–5.6). Transgender adults were more likely to report receiving poor quality health care (OR: 2.76, 95% CI: 1.64–4.65) and to be unfairly treated when receiving medical care (OR: 2.78, 95% CI:1.1–7.1).

**Table 4:** Adjusted odds of healthcare access and utilization for LGB (*n* = 73) compared to non-LGB/cisgender (*n* = 1830) (adjusted for age, gender, race, education, and income) and transgender (*n* = 25) compared to non-LGB/cisgender (*n* = 1830) (adjusted for age, race, education, and income) in Wisconsin from 2014 to 2016.

Outcome of interest	Odds ratio: LGB compared to non-LGB/cisgender (*n* = 73; *n* = 1830)	Odds ratio: transgender compared to non-LGB/cisgender (*n* = 73; *n* = 1830)	Percent of each group with the outcome of interest: LGB; transgender; non-LGB/cisgender
Taken less medicine than prescribed due to cost	2.13(0.82–5.7)	0.91(0.16–5.11)	23.61%; 12.00%; 11.59%
Usually see the same physician	0.62 (0.27–1.45)	0.61(0.23–1.57)	72.13%; 77.27%; 80.17%
Likelihood of not having a usual place of care	1.35(0.73–2.5)	1.71(0.36–8.15)	30.14%; 15.49%; 20.00%
Physical exam in the past year	1.12(0.6–2.08)	0.66(0.15–2.91)	23.61%; 16.67%; 17.98%
All medications covered by insurance	1.06(0.44–2.6)	0.61(0.26–1.41)	16.18%; 24.00%; 16.15%
All dental costs covered by insurance	0.91(0.53–1.5)	1.69(0.78–3.66)	62.12%; 63.94%; 50.00%
Preventative services not covered by insurance	1.89(1.10–3.23)⁎	1.92(0.72–5.1)	52.31%; 62.50%; 44.94%
Poor quality of care for routine physical exam	0.63(0.07–5.6)	4.63(0.88–24.34)+	1.82%; 10.00%; 3.55%
Anytime needed medical care but did not get it	2.08(0.59–7.26)	0.62(0.06–6.97)	19.18%; 8.00%; 7.94%
Delay in obtaining health care	2.17(1.07–4.4)⁎	1.08(0.29–4.1)	17.81%; 10.15%; 12.00%
Poor quality of healthcare	1.9(0.43–8.46)	2.76(1.64–4.65)⁎	8.20%; 9.10%; 3.88%
Lifetime experiences felt unfairly treated getting medical care	0.97(0.37–2.5)	2.78(1.10–7.10)⁎	18.87%; 21.74%; 10.4%

⁎ *p* < 0.05.

+ *p* < 0.10.

## 4 Discussion

### 4.1 Discussion of results on LGB health and health care access and utilization

Few population-based studies have published results on health care access and utilization for LGBT populations. The results of this study indicate that there are differences in how LGB and non-LGB/cisgender populations access and utilize health care services in Wisconsin, and this could be due to barriers to accessing appropriate health care, such as health care cost and coverage of preventative health services. For example, although LGB respondents were equally likely to have health insurance, they were less likely to have health insurance that covers the cost of preventative health care services and more likely to delay receiving health care compared to non-LGB/cisgender respondents.

### 4.2 Discussion of results on transgender health and health care access and utilization

Despite having a small sample with large confidence intervals, this study is among the first statewide population-based studies to document differences in health outcomes between transgender and cis respondents. Transgender respondents were over two times more likely to report poor or fair health status (95% CI: 1.34–3.7) and to have a chronic illness (95% CI:0.86–4.6), and almost three times more likely to receive poor quality health care (95% CI: 1.64–4.65) and to be unfairly treated when receiving health care (95% CI: 1.10–7.10). Although, the study sample was small and some of the confidence intervals are quite wide, these results are similar to those from the national 2015 Transgender Survey and the twenty-one state BRFSs, which both report a higher percentage of transgender people reporting poor or fair health compared to cisgender people (James and Herman, 2017; Kroenke et al., 2003). Our results add to the growing literature on how transgender people are more at risk for poor health outcomes and for receiving poor quality of healthcare (Kroenke et al., 2003; Lerner and Robles, 2017; Lombardi and Banik, 2016). Further, these barriers and risk factors suggest opportunities towards prevention and policies to reduce discrimination need to consider transgender adults as a particularly vulnerable population.

### 4.3 Implications

The results from this study in Wisconsin are important in that they support previous findings about LGBT health disparities from many regions around the United States (Institute of Medicine (US) Committee on Lesbian, Gay, Bisexual, and Transgender Health Issues and Research Gaps and Opportunities, 2011; Lovibond and Lovibond, 1995). The mounting evidence from population-based survey data support the need for federal and state public health and anti-discrimination policies to address LGBT health disparities (Graham et al., 2011). Discriminatory laws and stigma faced in medical care environments have discouraged LGBT people from revealing information about their sexual orientation and gender identity, making it difficult to sufficiently identify LGBT health disparities (Mayer et al., 2008). These discriminatory laws not only pose barriers to studying LGBT health disparities but also stem from stigma and bias against LGBT people, and these biases remain present in national and state policies that limit access to health care services to LGBT people and do not adequately protect the rights of LGBT people (Green et al., 2018; Meyer, 1995; Meyer and Wilson, 2009).

Even with a small number of respondents in this population-based study, there are significant findings that support the notion that health insurance access is another barrier to accessing high quality health care among LGBT adults in Wisconsin. LGB respondents were more likely not to have the cost of preventative services covered by their insurance, delayed getting care and to took less medicine than prescribed, and transgender respondents were more likely to receive poor quality of care and to experience unfair treatment when receive medical care. National and state policies contribute to limiting access to health insurance and coverage for health care services. Despite significant policy changes like the Affordable Care Act (ACA) and marriage equality (Meyer, 1995), disparities in access and coverage of care continue to exist in the United States. Nondiscrimination protections for health insurance and employment do not exist in most states, which prevents LGBT people from accessing and utilizing health care services to the same extent as non-LGBT people (Meyer, 1995). What's more, the Trump administration has continuously worked to dismantle LGBT health protections afforded under the Obama administration, such as rolling back anti-discrimination regulations under the ACA (Motwani and Fatehchehr, 2017), discouraging the use of words such as “evidence-based” or “transgender” in CDC budget documents (Movement Advancement Project, 2018), and proposing that federal agencies define sex as an immutable category based on birth genitalia or chromosomes (National Center for Transgender Equality, 2017).

In addition to the systemic barriers discussed above, provider discrimination and poor provider training may also prevent LGBT people from accessing necessary and appropriate health care (Conron et al., 2010; Lombardi and Banik, 2016; Patterson et al., 2017). Data from this study are in agreement with previous research in other regions of the United States documenting a higher risk for poor physical and mental health due to a combination of factors related to discrimination, stigma and internalized homophobia LGBT populations (Institute of Medicine (US) Committee on Lesbian, Gay, Bisexual, and Transgender Health Issues and Research Gaps and Opportunities, 2011; Lovibond and Lovibond, 1995). All the while LGBT populations are less likely to access and utilize health care services due to cost, not being offered appropriate preventative health screenings, or being refused care or coverage for care (Cornelius and Carrick, 2015; Patterson et al., 2017; Ranji and Beamesderfer, 2018). Further, fear of discrimination in the medical care setting not only prevents transgender people from accessing health care but also influences whether they disclose their gender identity to their provider, which is an additional barrier to receiving appropriate health care (Mayer et al., 2008).

These gaps in health outcomes and health care quality demonstrate that it is not sufficient to simply improve access to affordable health care. To entirely close the gap, improved provider training on LGBT health and health disparities are necessary to extend health care and high quality, appropriate health care to all LGBT populations (Gorman, 2016).

### 4.4 Limitations

The analysis was limited by the questions asked about sexual and gender identity in the SHOW questionnaire and by the size of the survey sample. As with other population-based studies, the SHOW questionnaire includes some questions on sexual orientation and gender identity, but these questions do not follow the validated, best practice recommendations of the Williams Institute (Braveman et al., 2010; Centers for Disease Control and Prevention, 2017; Cohen, 2017; Sabin et al., 2015). By not including all three questions about the three dimensions of sexual orientation and the two-step approach to asking about gender identity, the SHOW questionnaire most likely underestimates the percentage of respondents who are LGBT (Centers for Disease Control and Prevention, 2017; Saewyc et al., 2004). Further, there are inconsistencies in the SHOW questionnaire with regard to time when the respondent identified with a particular gender identity or sexual orientation. The question about gender identity asks about current or past gender identity, but the sexual orientation question does not have a reference to the time (current, past, or both). The recommended practice for population-based surveys is to ask for the respondent to answer how they describe their sexual orientation and gender identity without a reference to current or past identity (Cohen, 2017).

A second limitation of the survey is that reproductive health screenings are only asked of those who report their gender as female and prostate screenings are only asked of individuals who report their gender as male. Using gender as an indicator of whether a participant is eligible to answer these questions, rather than an indicator based on sex assigned at birth, prevents the survey from capturing how these services are used by transgender respondents. These are essential measures given the known barriers that prevent transgender individuals from receive appropriate preventative health screenings (Conron et al., 2010; Cornelius and Carrick, 2015; Mayer et al., 2008; The GenIUSS Group and Herman, 2014).

Third, there are limitations to using a randomly sampled cross-sectional survey. First, the LGBT populations make up only small proportion of the population, so the survey sample of LGBT respondents is too small to estimate state-level prevalence of various health behavior and health care access and utilization indicators for LGBT sub-populations. In future studies, it will be important to make use of other sampling techniques, such as convenience sampling, in order to increase the number of LGBT respondents in the survey sample (The Williams Institute and Badgett, 2009). Second, the cross-sectional survey data can only estimate current health care access and utilization and health outcomes. Without longitudinal data, we are unable to use these data to understand how these factors might contribute to LGBT disparities in health outcomes.

## 5 Conclusions

The goal of the CDC's Healthy People 2020 is to improve health by eliminating health disparities and promoting health equity. The results of this study are important because they add to the growing literature on LGBT health disparities and barriers to accessing and utilizing health care services. However, as we approach 2020, it becomes clear that LGBT health disparities still exist in the United States, and great changes in policy and healthcare delivery are still needed to achieve health equity for LGBT populations. Given the current knowledge of these health disparities and of the barriers that prevent LGBT populations from accessing and utilizing health care resources, steps need to be taken at many levels to reach the goals set by Healthy People 2020.

One of the next steps we need to take to begin to reduce these health disparities is to conduct more research that focuses on how health care is provided to LGBT populations at the health care system and provider levels and on how to design and implement interventions to improve provider training in serving LGBT populations. To take this next step, we need to both standardize how we measure LGBT populations and improve how we conduct population-based health survey research. First, population-based surveys need to include the recommended best practice questions published by the Williams Institute to identify LGBT respondents and questions on patient experience with providers and the health care system to better understand the health care services needs of LGBT people. Including these questions is essential for researchers, providers and policy makers to better understand the barriers to receiving necessary and appropriate health care. Second, often LGBT population samples are small, even in state and national population-based studies, which limit our ability to study these populations. This could be addressed by over-sampling LGBT populations by targeting neighborhoods are areas with larger populations of LGBT people, which has been used in other population-based studies to capture a larger sample of LGBT people (The Williams Institute and Badgett, 2009). These changes to population-based survey questions are necessary to assess the patient experience so that these data can be used to design health care systems and provider training programs that are centered on improving health care services and health outcomes for LGBT populations.

## Conflict of interest

None.
